# Considerations for the use of porcine organ donation models in preclinical organ donor intervention research

**DOI:** 10.1002/ame2.12411

**Published:** 2024-04-30

**Authors:** Frazer I. Heinis, Shaheed Merani, Nicholas W. Markin, Kim F. Duncan, Michael J. Moulton, Lance Fristoe, William E. Thorell, Raechel A. Sherrick, Tami R. Wells, Matthew T. Andrews, Marian Urban

**Affiliations:** ^1^ School of Natural Resources Institute of Agriculture and Natural Resources, University of Nebraska‐Lincoln Lincoln Nebraska USA; ^2^ Division of Transplantation and Vascular Surgery, Department of Surgery University of Nebraska Medical Center Omaha Nebraska USA; ^3^ Department of Anesthesiology University of Nebraska Medical Center Omaha Nebraska USA; ^4^ Division of Cardiothoracic Surgery, Department of Surgery University of Nebraska Medical Center Omaha Nebraska USA; ^5^ Clinical Perfusion Nebraska Medicine‐Nebraska Medical Center Omaha Nebraska USA; ^6^ Department of Neurosurgery University of Nebraska Medical Center Omaha Nebraska USA; ^7^ Nutrition and Health Sciences, College of Education and Human Sciences University of Nebraska‐Lincoln Lincoln Nebraska USA; ^8^ Department of Comparative Medicine University of Nebraska Medical Center Omaha Nebraska USA

**Keywords:** animal model, brain death, circulatory death, organ transplantation

## Abstract

Use of animal models in preclinical transplant research is essential to the optimization of human allografts for clinical transplantation. Animal models of organ donation and preservation help to advance and improve technical elements of solid organ recovery and facilitate research of ischemia–reperfusion injury, organ preservation strategies, and future donor‐based interventions. Important considerations include cost, public opinion regarding the conduct of animal research, translational value, and relevance of the animal model for clinical practice. We present an overview of two porcine models of organ donation: donation following brain death (DBD) and donation following circulatory death (DCD). The cardiovascular anatomy and physiology of pigs closely resembles those of humans, making this species the most appropriate for pre‐clinical research. Pigs are also considered a potential source of organs for human heart and kidney xenotransplantation. It is imperative to minimize animal loss during procedures that are surgically complex. We present our experience with these models and describe in detail the use cases, procedural approach, challenges, alternatives, and limitations of each model.

## Graphical abstract

Large animal models are essential for experimental progress in organ transplant procedures and donor organ interventions. This review provides a summary of two major organ donation procedures, a detailed methods description and pharmacopeia for these procedures, an overview of existing research progress in these areas, and our experience in potential challenges, procedural considerations, and alternative methods relating to porcine organ donor research.

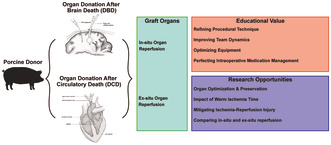

## 1 INTRODUCTION

Transplantation is the most efficient therapy for end‐stage organ failure. However, the unavailability of adequate organs for transplantation to meet existing demand has resulted in major organ shortage crises.^1^ Despite the growing gap between the supply of donor organs and increasing number of recipients on the transplant waiting lists, data show that only a fraction of offered organs are ultimately accepted for transplantation.^2^, ^3^ Expanding the donor pool by increasing utilization of marginal organs together with optimizing the preservation methods for out‐of‐body organ transport are key components of the strategy for ameliorating the organ shortage. As the need for transplants continues to outpace the availability of brain dead donors, the use of organs, including heart, lungs, liver and kidney from donors after circulatory death (DCD) has been established as a source of additional organs in both adult and pediatric patient populations.^4^, ^5^, ^6^ Despite clinical success of DCD from Maastricht category III donors, there remain concerns about the effect of warm ischemia on post‐transplantation graft performance.^7^ The upper limit of warm ischemia duration along with optimal recovery technique and preservation strategy for DCD organs are currently not known and require further research.^8^, ^9^, ^10^, ^11^

Animal models play an indispensable role in enabling biomedical studies to address these aims.^12^ In the context of transplantation research, porcine models are an essential research tool to optimize organ procurement practices and serve important roles in procedural refinement, mechanistic investigation, and surgical team cohesion (Table 1).^13^, ^14^, ^15^, ^16^ The reproducibility of pig models has also permitted investigation into optimal donor organ preservation.^17^, ^18^

**TABLE 1:** Large animal experimental model utility in transplantation research.

*Donation after brain death (DBD)*
Study the impact of brain death on the quality of donor organs Evaluate different preservation strategies for DBD organs
*Donation after circulatory death (DCD)*
Study the impact of warm ischemia on the quality of DCD organs Evaluate different recovery techniques, interventions, and preservation strategies to improve DCD organ quality Facilitate and improve recovery team technical skills, communication, and cooperation during clinical DCD organ recoveries

Studies using large animal models for donor organ research can be grouped into three distinct categories. In the first category (Table 2A) are studies evaluating hemodynamic, metabolic, and hormonal changes associated with brain death and the effects of brain death on donor organ function^19^, ^20^, ^21^, ^22^, ^23^, ^24^, ^25^ or pharmacologic interventions to mitigate the adverse effects of brain death.^26^, ^27^ Similarly, DCD large animal models have been developed to evaluate the impact of warm ischemia on organ donor function.^28^, ^29^, ^30^ In the second category (Table 2B) are studies that evaluate experimental methods of DBD graft preservation and technical and pharmacological modifications of DCD graft recovery.^17^, ^31^, ^32^, ^33^, ^34^, ^35^, ^36^, ^37^, ^38^, ^39^, ^40^, ^41^ In the third category (Table 2C) are studies that combine the use of large animal models for both organ donation and organ transplantation.^42^, ^43^, ^44^, ^45^, ^46^, ^47^, ^48^, ^49^, ^50^, ^51^

**TABLE 2A:** Studies using large animal models in pre‐clinical donor organ research evaluating hemodynamic, metabolic, and hormonal changes during brain death and circulatory death.

Author, journal, date	Model	Death induction	Study group	Main findings
Bittner et al.^19^ *J Heart Lung Transplant* 1995	DBD	Inflation of a subdurally placed balloon	10 dogs	Cushing reflex, hyperdynamic state, catecholamine storm, vasopressin and adrenocorticotropic hormone cessation, total cerebral necrosis, and diabetes insipidus were consistent findings
Sebening et al.^20^ *Eur J Cardiothorac Surg* 1995	DBD	Inflation of a subdurally placed balloon	10 dogs	Acutely induced irreversible intracranial hypertension leads to multifactorial hemodynamic and hormonal changes
Bittner et al.^21^ *Circulation* 1995	DBD	Inflation of a subdurally placed balloon	17 dogs	BD causes significant systolic biventricular dysfunction. The loss of ventricular function after BD was more prominent in the right ventricle
Bittner et al.^22^ *Chest* 1995	DBD	Inflation of a subdurally placed balloon	20 dogs	BD causes significant changes in pulmonary vascular hemodynamics
Chen et al.^23^ *Crit care Med* 1996	DBD	Inflation of a subdurally placed balloon	10 dogs	Significant decreases in the circulating concentrations of stress hormones, as well as hemodynamic instability, occurred after brain death
Golling et al.^24^ *Transplantation* 2003	DBD	Inflation of a subdurally placed balloon	16 pigs	During brain death, liver‐specific parameters (portal venous flow, microperfusion, aspartate aminotransferase activity, ENC, and hepatic oxidative stress) were compromised, independent of the hemodynamic status. Therefore, the systemic hemodynamic status does not reflect the functional status of the liver during BD
Purins et al.^25^ *Critic Care Med* 2011	DBD	Inflation of an epidural balloon catheter.	6 pigs	The standardized brain death model designed in pigs simulates the clinical development of brain death in humans with a classic pressure‐volume response and systemic cardiovascular reactions
Belhaj et al.^27^ *J Heart Lung Transplant* 2016	DBD	Intracranial infusion of blood	19 pigs	Brain death‐induced RV dysfunction is associated with RV activation of inflammation and apoptosis and is partly limited by methylprednisolone
Belhaj et al.^26^ *Am J Respir Crit Care Med* 2022	DBD	Intracranial infusion of blood	17 pigs	Immunomodulation through tacrolimus pretreatment prevented pulmonary capillary hypertension as well as the activation of inflammatory and apoptotic processes in the lungs after brain death
Iyer et al.^28^ *Am J Transplant* 2014	DCD	Hypoxic circulatory arrest followed by 20,30, and 40 min of warm ischemia.	30 pigs	DCD hearts with </=30‐min WIT may be suitable for transplantation and warrant assessment in a transplant model
White et al.^29^ *Am J Transplant* 2016	DCD	Hypoxic circulatory arrest followed by 20 min of warm ischemia.	19 pigs	Hypoxic pulmonary vasoconstriction and a profound catecholamine surge occur following WLST that result in distension of the RV
Soussi et al.^30^ *Biomed res Int* 2019	DCD	Through a combination of pharmacological and technical approaches, MAP was decreased to below 45 mmHg and maintain the animal in this state for 90 min	8 pigs	Development of a porcine model of donation after circulatory death kidney transplantation

Abbreviations: BD, brain death; DBD, donation after brain death; DCD, donation after cardiac death; ENC, endotoxin‐neutralizing capacity; MAP, mean arterial pressure; RV, right ventricle; WIT, warm ischemic time; WLST, withdrawal of life sustaining treatment.

**TABLE 2B:** Studies using large animal models in pre‐clinical donor organ research evaluating procurement techniques and preservation methods.

Author, journal, date	Model	Death induction	Study group	Main findings
Michel et al.^17^ *Ann Transplant* 2014	DBD	Potassium induced cardiac arrest	6 pig hearts stored in hypothermic oxygenated perfusion 6 pig hearts stored in ice	Hypothermic pulsatile perfusion of donor hearts leads to a better‐preserved cell structure compared to the conventional cold storage method
Schuele et al.^31^ *J Heart Lung Transplant* 2003	DCD	Hypoxic circulatory arrest followed by 7 ± 2 min of warm ischemia followed by 90 min of cold ischemia	20 pigs	Cariporide as an additive to UW solution improves functional recovery and decreases myocardial damage in DCD hearts protected with an *in situ* perfusion technique
Osaki et al.^32^ *Ann Thorac Surg* 2006	DCD	Hypoxic circulatory arrest followed by 30 min of warm ischemia	18 pigs	The best recovery was observed in the DCD hearts resuscitated by continuous myocardial perfusion when the initial controlled reperfusion with lukewarm blood cardioplegic solution at 40 mmHg lasted for 20 min
Kotani et al.^33^ *J Thorac Cardiovasc Surg* 2007	DCD	Hypoxic circulatory arrest followed by 30 min of warm ischemia	12 pigs	Antioxidant therapy with MCI‐186 at the initial reperfusion is essential to successful resuscitation of DCD hearts by continuous myocardial perfusion
Allain et al.^34^ *Transplant Res* 2014	DCD	Potassium induced cardiac arrest followed by 30 min of warm ischemia	6 pigs	Development of A‐NRP protocol
White et al.^35^ *Ann Thorac Surg* 2017	DCD	Hypoxic circulatory arrest followed by 15 min of warm ischemia	41 pigs	Initial reperfusion of hearts donated after circulatory death with a hypocalcemic and moderately acidic cardioplegia minimizes edema and optimizes functional recovery during subsequent ex vivo heart perfusion
Guariento et al.^36^ *J Heart Lung Transplant* 2020	DCD	Hypoxic circulatory arrest followed by 20 min of warm ischemia	29 pigs	Mitochondrial transplantation significantly preserves myocardial function and oxygen consumption in DCD hearts
Saemann et al.^37^ *Antioxidants* 2022	DCD	Hypoxic circulatory arrest followed by 30 min of warm ischemia.	10 pigs	The use of a cytokine adsorption device during *ex‐situ* perfusion counteracts preload‐dependent microvascular dysfunction and preserves the endothelium by preventing oxidative stress and IRI of coronary arterioles of DCD hearts.
Dalsgaard et al.^38^ *Transplantation* 2022	DCD	Hypoxic circulatory arrest followed by 8 min of warm ischemia and 30 min of TA‐NRP	16 pigs	Clamping of the arch vessels during TA‐NRP halts cerebral circulation, ensuring the permanent cessation of brain function and maintaining the determination of death in DCD
Khalil et al.^39^ *J Vis Exp* 2022	DCD	Hypoxic circulatory arrest followed by warm ischemia and 30 min–3 h of TA‐NRP	Pigs	Development of a porcine model of DCD heart recovery with TA‐NRP technique
Moeslund et al.^40^ *Transplantation* 2022	DCD	Hypoxic circulatory arrest followed 15 min of warm ischemia and 50 min of TA‐NRP	19 pigs	All hearts weaned from NRP showed acceptable hemodynamic function for transplantation. Hearts exposed to low oxygenation (FiO2 0.021) showed more myocardial damage and showed poorer contractile performance than hearts reperfused with high oxygenation (FiO2 1.0)
Blondeel et al.^41^ *J Vis Exp* 2023	DCD	Clamping the thoracic aorta followed by 60 min of warm ischemia	Pigs	Development of a porcine normothermic isolated liver perfusion protocol

Abbreviations: A‐NRP, abdominal normothermic regional perfusion; IRI, ischemia–reperfusion injury; TA‐NRP, thoracoabdominal normothermic regional perfusion; UW, University of Wisconsin Solution.

**TABLE 2C:** Studies using large animal models in pre‐clinical donor organ research evaluating post transplantation outcomes.

Author, journal, date	Model	Death induction	Study group	Main findings
Bittner et al.^42^ *Ann Thorac Surg 1995*	DBD	Inflation of a subdurally placed balloon	34 dog heart transplantations	Brain death causes a significant loss of right and left ventricular function. These injuries are greater in the right ventricle and may contribute to early right ventricular failure after transplantation. Brain death and cardiac preservation interact significantly to impair right ventricular function further
Kerforne et al.^43^ *Int J Mol Sci* 2019	DBD	Inflation of a subdurally placed balloon	12 pig kidney transplantations	Resumption of early kidney graft function post‐transplant was better in the rapidly generated brain death group in comparison to the slow generated group
Abbasi et al.^44^ *Front Immunol* 2020	DBD	Inflation of a subdurally placed balloon	26 pig kidney transplantations	Oral preconditioning with Cyclosporine or Everolimus is feasible in donation after brain death pig kidney transplantation and reduces the expression of TNF‐alpha.
Martin et al.^45^ *J Thorac Cardiovasc Surg* 2003	DCD/DBD	Exsanguination followed by 30 min of warm ischemia (DCD) Potassium induced cardiac arrest followed by 30 min of cold ischemia (DBD)	6 pig DCD heart transplantations 6 pig DBD heart transplantations	Recovery of donor hearts from DCD donors was comparable with recovery of organs harvested from DBD donors if the blood cardioplegia was supplemented with a sodium‐hydrogen exchange inhibitor and adenosine in the DCD group
Ali et al.^46^ *Am J Transplant* 2011	DCD/DBD	Hypoxic cardiac arrest followed by 15 min of warm ischemia (DCD) Inflation of a subdurally placed balloon (DBD)	5 pig DCD heart transplantations 5 pig DBD heart transplantations	After transplantation, cardiac function was comparable between DBD and DCD groups
Ribeiro et al.^47^ *Circ Heart Fail* 2019	DCD/DBD	Hypoxic circulatory arrest followed by warm ischemia and TA‐NRP (DCD) Potassium induced cardiac arrest (DBD)	4 pigs DBD transplantations 5 pigs DCD transplantations of hearts stored in static storage with histidine‐tryptophan‐ketoglutarate (HTK) at 4 °C 5 pigs DCD transplantations of hearts stored in static storage with histidine‐tryptophan‐ketoglutarate (HTK) at 21 °C	DCD hearts stored at 4 °C using a standard preservation solution demonstrated comparable post‐transplantation myocardial function to standard controls
Osaki et al.^48^ *J Heart Lung Transplant* 2009	DCD	Hypoxic circulatory arrest (*N* = 6) followed by 30 min of warm ischemia Exsanguination (*N* = 6) followed by 30 min of warm ischemia	12 pigs	Cardiac arrest with circulatory load by asphyxiation caused more myocardial damage than unloaded arrest in DCD hearts
White et al.^49^ *J Heart Lung Transplant* 2013	DCD	Hypoxic circulatory arrest followed by 15 min of warm ischemia	17 pigs	Initial reperfusion with a tepid adenosine‐lidocaine cardioplegia and continuous myocardial perfusion minimizes myocardial injury and improves short‐term post‐transplant function of DCD hearts
Tillet et al.^50^ *Transl Res* 2016	DCD	Renal artery clamping followed by 60 min of warm ischemia and heterotopic kidney transplantation	24 pigs	Using anti‐Xa/IIa during kidney flush and preservation reduced thrombin generation at revascularization, improved early kidney graft functional recovery, and decreased chronic lesions
Goerlich et al.^51^ *Front Immunol* 2021	N/A	Cardioplegia induced cardiac arrest	8 pigs to baboon heart xenotransplantations	Blood cardioplegia induction, alone or followed by non‐ischemic storage (hypothermic oxygenated perfusion), reduced the incidence of peri‐operative cardiac xenograft dysfunction and improved graft function and survival, relative to traditional crystalloid cardioplegia‐static cold storage alone

## 2 OVERVIEW OF LARGE ANIMAL MODELS OF ORGAN DONATION FOR RESEARCH

### 2.1 Donation after brain death

Most large animal models of DBD involve the use of a subdural balloon to elevate intracranial pressure (ICP), thereby compromising brain perfusion and causing global brain and brainstem ischemia and herniation. Cushing reflex, hyperdynamic state, catecholamine storm, vasopressin and adrenocorticotropic hormone cessation, total cerebral necrosis, and diabetes insipidus are consistent findings after intracranial pressure rises above brain perfusion pressure for at least 30 min.^19^, ^27^, ^44^

### 2.2 Donation after circulatory death

The most clinically relevant animal DCD model is based on asphyxiation and circulatory arrest subsequent to the withdrawal of mechanical ventilation in a paralyzed animal.^39^ To a certain degree, the model allows for control over the duration of hypoxic and hypotensive warm ischemia and thus facilitates studies focused on the impact of these intervals on graft injury and post‐reperfusion performance. This model can also be used to produce allografts for *in‐situ* (inside of the body) reanimation of multiple organs with either abdominal or thoracoabdominal normothermic perfusion and *ex‐situ* (outside of the body) single organ machine perfusion after direct procurement.^34^, ^40^, ^41^, ^47^, ^52^ These platforms enable organs to be evaluated after a period of ischemia, and facilitate further research aimed at reanimation and preservation of DCD allografts.

## 3 PROCEDURAL DESCRIPTION OF ANIMAL MODELS

### 3.1 Veterinary care, anesthesia induction, and common instrumentation

All animal work needs approval from the Institutional Animal Care and Use Committee before commencing the experiments. For both DBD and DCD procedures, veterinary care, anesthesia induction, and certain instrumentation and vascular access lines are similar. The common initial steps are described here, followed by DBD‐ and DCD‐specific protocols. Essential and optional measurements are summarized in Table 3. Vascular access is summarized in Table 4. A complete pharmacopeia for DBD and DCD procedures is provided in Table 5.

1. Pigs of both sexes from an approved commercial vendor can be used. Body weight can range widely, from 20 to 60 kg, depending on experimental design.
2. Thirty minutes before surgery, intramuscular atropine (0.05 mg/kg), tiletamine/zolazepam 1:1 ratio (Telazol, 4.4–5 mg/kg), ketamine (2.2–2.5 mg/kg), and xylazine (2.2–2.5 mg/kg) are administered for premedication (Table 5).
3. Animals are placed in the prone position. General anesthesia is induced with mask‐ inhaled isoflurane to effect followed by oral intubation and mechanical ventilation with inhaled isoflurane 1%–4% (Table 5). Respiratory rate is adjusted to target end‐tidal CO_2_ of 35–40 mmHg and oxygen saturation above 95%.
4. Animals are placed in a lateral position to place five ECG electrodes: left shoulder (black); right shoulder (white); left lower extremity (red); left lateral aspect of chest (brown); and near right shoulder (green; grounding). Pulse oximetry is obtained from the ear, snout, lip, or tongue.
5. A 20G catheter is placed in the ear vein for peripheral IV (Table 4). Animals are placed in the supine position. Using the Seldinger technique with ultrasound guidance (Butterfly iQ+ Handheld Ultrasound, Butterfly Network, Palo Alto, CA, USA) an arterial catheter is inserted percutaneously in the left common femoral artery for invasive blood pressure monitoring and sampling. In the same manner, a multi‐lumen catheter is inserted percutaneously into the superficial femoral vein for sampling and drug delivery (Table 4).
6. Vital signs are monitored, and data recorded using dedicated software (VitalRecorder, Vital DB, Seoul, South Korea).
7. Normal saline is infused following the 4/2/1 rule (4 mL/kg/h for the first 10 kg, 2 mL/kg/h for the next 10 kg, and 1 mL/kg/h for each kg after the first 20 kg).

**TABLE 3:** Essential and optional monitoring for porcine DBD and DCD models.

Essential monitoring	Optional measurements
*DBD monitoring*	
Invasive arterial pressure Intracranial pressure 2‐lead Electrocardiogram (ECG) Pulse oximetry End tidal CO_2_	Continuous measurement of cardiac output Central venous pressure Pulmonary artery pressure Pulmonary capillary wedge pressure Rectal temperature Mixed venous saturation
*DCD monitoring*	
Invasive arterial pressure Central Venous pressure 2‐lead Electrocardiogram (ECG) Pulse oximetry End tidal CO_2_	Continuous measurement of cardiac output Pulmonary artery pressure Pulmonary capillary wedge pressure Rectal temperature Mixed venous saturation Continuous evaluation of cardiac performance using pressure volume loops

**TABLE 4:** Vascular access catheters for porcine organ recovery models.

Vascular access	Location	Catheter	Manufacturer	Purpose
*Vascular access for both DBD and DCD procedures*				
Arterial	Common femoral artery	Arrow® Seldinger Arterial catheter	Teleflex Inc, Wayne, PA	Monitoring, sampling
		20G × 12 cm × 0.025″		
Venous	Superficial femoral vein	3 lumen Arrowg+ard Blue®	Teleflex Inc, Wayne, PA	Sampling, drug delivery
		8.5Fr × 16 cm × 0.032″		
Venous	Ear vein	20G Angiocath™	BD, Franklin Lakes, NJ	Maintenance saline infusion
*Additional vascular access for experimental DCD procedures*				
Arterial	Right common carotid artery	Pinnacle® Introducer Sheath	Terumo Interventional Systems, Ann Arbor, MI	Guide for PV loop catheter
		8Fr × 10 cm		
Arterial	LV via Right common carotid artery	Pressure‐volume impedance catheter	Transonic Systems, Ithaca, NY	PV loop catheter
		7 Fr		
Venous	Superior vena cava	2 lumen Arrowg+ard Blue®	Teleflex Inc, Wayne, PA	Sampling, drug delivery, pulmonary artery catheter
		14Fr × 11.5 cm × 0.032″		
Venous	External jugular vein	2 lumen Arrowg+ard Blue®	Teleflex Inc, Wayne, PA	Sampling, drug delivery, sheath for Swan‐Ganz
		14Fr × 11.5 cm × 0.032″		

**TABLE 5:** Pharmacopeia for porcine organ donation experimental models.

Drug	Dose	Route of administration	Time of administration	Responsible personnel	DBD or DCD
Atropine	0.05 mg/kg	Intramuscular, bolus	30 min before procedure	Veterinarian	Both
Telazol	4.4–5 mg/kg	Intramuscular, bolus	30 min before procedure	Veterinarian	Both
Ketamine	2.2–2.5 mg/kg	Intramuscular, bolus	30 min before procedure	Veterinarian	Both
Xylazine	2.2–2.5 mg/kg	Intramuscular, bolus	30 min before procedure	Veterinarian	Both
Isoflurane	1%–4%	Inhalational, continuous	During anesthesia	Veterinarian	Both
Normal saline	4/2/1 rule	Intravenous, continuous	During anesthesia	Veterinarian	Both
Rocuronium	1.5 mg/kg	Intravenous, bolus	Prior to intracranial balloon inflation	Veterinarian	DBD
Midazolam	0.5 mg/kg	Intravenous, bolus	Prior to intracranial balloon inflation	Veterinarian	DBD
Vasopressin	0.02–0.04 units/min	Intravenous, continuous	After brain/circulatory death	Anesthesiologist	Both
Norepinephrine	0.02–0.06 mcg/kg/min	Intravenous, continuous	After brain/circulatory death	Anesthesiologist	Both
Normal saline	10 mL/kg	Intravenous, boluses as needed	After brain/circulatory death	Anesthesiologist	Both
Heparin	300 units/kg	Intravenous, bolus	Prior to organ recovery	Anesthesiologist	Both
Lidocaine	50 mg	Intravenous, bolus	Before sternotomy	Anesthesiologist	DCD
Propofol	14–20 mg/kg/h	Intravenous, continuous	Before discontinuation of ventilatory support	Anesthesiologist	DCD
Vecuronium	1 mg/kg	Intravenous, bolus	Before discontinuation of ventilatory support	Anesthesiologist	DCD

### 3.2 Donation after brain death

**FIGURE 1:**
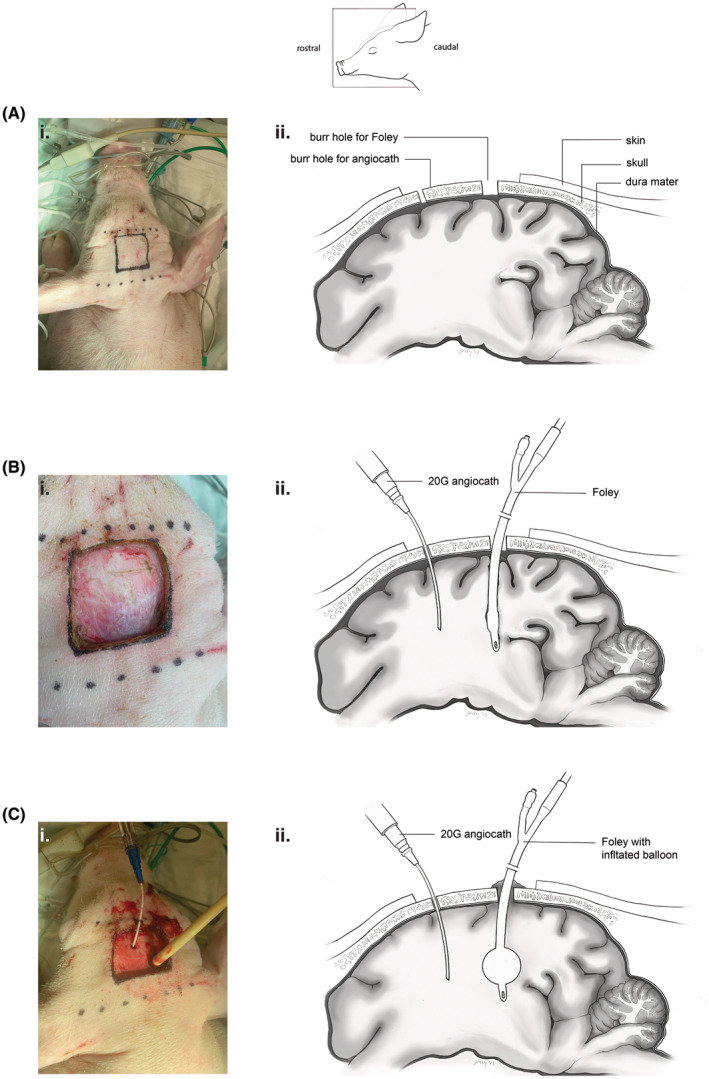
Catheters and craniotomy landmarks for DBD procedure. (Ai) External craniotomy landmarks on dorsal skull. (Aii) Parasagittal cross‐section of burr hole locations relative to the brain parenchyma. (Bi) 5 × 5 cm skin flap is raised to identify the coronal and sagittal sutures. Two contralateral burr holes are drilled. One burr hole is widened to admit the 14 Fr balloon‐tipped Foley catheter, and the other is sized to admit a 20G angiocath for ICP measurement. (Bii) Parasagittal view of the 20G angiocath ICP monitor and Foley placement prior to Foley inflation. (Ci) Dorsal view of inserted angiocath and Foley catheters. (Cii) the Foley balloon tip is inflated with water until an appropriate ICP and hemodynamic response is observed. Sulci are compressed. Gray matter may be observed extruding from the Foley burr hole site.

1. After induction of general anesthesia, intubation, insertion of percutaneous arterial and venous lines, and placement of non‐invasive monitoring, the animals are positioned prone. Care is taken to avoid displacing vascular access and non‐invasive monitors as the animal is repositioned.
2. Rocuronium (1.5 mg/kg) and midazolam (0.5 mg/kg) are administered prior to craniotomy (Table 5) to suppress potential seizures and associated movement.
3. A 5 × 5 cm skin flap is excised from the dorsal skull (Figure 1A,B). Coronal and sagittal sutures are identified (Figure 1B), and two burr holes are drilled through the skull in the lateral frontotemporal regions using a rotary drill (Figure 1B,C). One burr hole is enlarged to approximately 6 mm to accommodate the balloon of a Foley catheter.
4. A 20G angiocath is inserted through the smaller burr hole for ICP measurement. The catheter must be flushed to ensure the appropriate tracing for accurate ICP measurement. A 14‐Fr 2‐way balloon‐tipped Foley catheter is introduced through the larger burr hole and positioned inside the subdural/subarachnoid space.
5. The Foley balloon is gradually inflated with water to elevate ICP above mean arterial pressure (MAP), typically in 5 cc increments with a 5‐min pause to assess physiological response.
6. Brainstem death (BSD) is established after a 30‐min interval when ICP pressure is maintained above MAP. This is typically preceded by transient hypertension and tachycardia (Cushing response). Absence of a Cushing response in the presence of continuously elevated ICP confirms brainstem death.
7. Animals often require vasoactive management following BSD due to marked hypotension. Our initial intervention is vasopressin infusion at 0.02 U/min and saline boluses of 10 mL/kg (Figure 3). Additionally, vasopressin infusion may be increased to 0.04 U/min and norepinephrine infusion ranging from 0.02 to 0.06 mcg/kg/min may be initiated as necessary.
8. After brain death is confirmed, the animals are turned supine for organ recovery.

### 3.3 Donation after circulatory death

**FIGURE 2:**
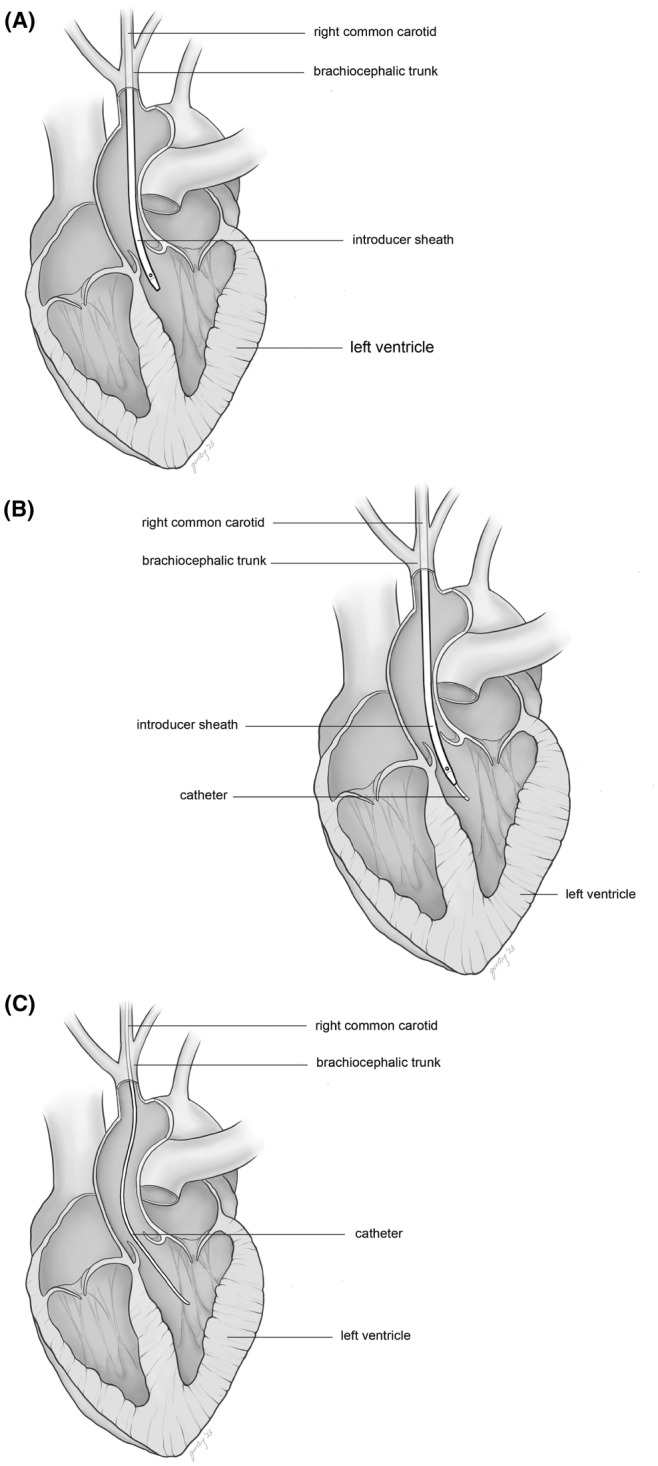
LV catheter placement for DCD procedure. (A) Four‐chamber cross section view of porcine heart. An 8 Fr introducer sheath fully enclosing a 7 Fr pressure conductance catheter has been advanced from the right common carotid artery past the aortic valve. (B) The 7 Fr pressure conductance catheter has been advanced from the introducer sheath into the left ventricle. (C) The introducer sheath is withdrawn to leave the pressure conductance catheter positioned in the left ventricle.

1. The porcine model of circulatory death is based on the hypoxic circulatory arrest after controlled withdrawal from respiratory support in a paralyzed animal. Essential monitoring and optional measurements during withdrawal in DCD model are described in Table 3B. The rationale for essential monitoring is to define the time intervals for hypoxic and hypotensive warm ischemia periods. Optional measurements can provide additional invasive hemodynamic data.
2. After induction of general anesthesia and obtaining femoral vascular access (as described in Section 2.1: Veterinary Care, Anesthesia Induction, and Common Instrumentation) an 8 Fr sheath is inserted percutaneously under ultrasound guidance in the right common carotid artery. A 7 Fr Pressure‐volume impedance catheter (Transonic Systems, Ithaca, NY, USA) is inserted in left ventricle (LV) via the sheath in the right common carotid artery (Figure 2). Transesophageal ultrasound (Phillips iE33 Ultrasound with X7‐2t TEE Probe) is used to confirm the correct placement of the cannulae/catheters and to evaluate cardiac function after manipulation.
3. Lidocaine (50 mg IV bolus) is administered before sternotomy to prevent ventricular arrhythmias. A median sternotomy is performed. The pericardial sac is opened, and a pericardial well is created with stay sutures using 0‐silk.
4. A multi‐lumen catheter (14 Fr) is inserted under direct visualization in the right atrium through the superior vena cava. The catheter is used for pressure monitoring and drug delivery. An 8.5 Fr Swan‐Ganz pulmonary arterial catheter with mixed venous oxygen saturation (Edwards Lifescience, Irvine, CA, USA) is inserted through the distal port of the multilumen catheter and advanced into the PA.
5. Heparin (Table 5) is administered immediately prior to planned discontinuation from ventilatory support. Anesthesia with inhalation isoflurane is switched to an IV continuous infusion of Propofol (14–20 mg/kg/h). A bolus of vecuronium (1 mg/kg IV) is delivered, followed by discontinuation of mechanical ventilation. The breathing circuit from the anesthesia machine is then disconnected to prevent apneic oxygenation.
6. Hypoxic cardiac arrest is declared when central venous pressure equals mean arterial pressure and mechanical asystole is noted. These events are commonly preceded by a brief elevation of heart rate and blood pressure which is presumed to be the result of perimortem release of endogenous catecholamines.
7. The time interval from the withdrawal of ventilatory support to cardiac arrest varies from animal to animal. After death declaration, there may be a variable period of warm ischemia added before organs are removed to standardize the total length of warm ischemia for all study animals. The method of organ procurement is dependent on whether *ex‐situ* or *in‐situ* organ resuscitation is planned.
8. If *ex‐situ* reperfusion is the intended recovery method, median sternotomy and laparotomy are performed, organs are flushed with the organ specific cold storage solution, removed from the donor, and instrumented on *ex‐situ* apparatus.
9. *In‐situ* reperfusion requires placement of the drainage (venous) and return (arterial) cannulae for the extracorporeal mechanical circulatory support to drive the circulation with oxygenated blood in the absence of the native heart and lung function. The venous cannula is placed into the inferior vena cava through right atrial appendage, the arterial cannula is placed in the ascending aorta. The brain is excluded from circulation by clamping the supra‐aortic vessels before the perfusion is initiated. As opposed to real clinical practice when instrumentation is only permitted after death, in the animal model it is advisable to cannulate great vessels before ventilation is stopped. This promotes animal stability and avoids animal loss from surgical misadventures that may occur because of the haste to initiate extracorporeal circulation in effort to minimize the time sensitive organ damage.

## 4 LIMITATIONS AND CHALLENGES OF ANIMAL MODELS

### 4.1 Donation after brain death

The model of brain death based on elevating intracranial pressure using the balloon of a Foley catheter inflated inside the skull is very reproducible and technically not demanding. The required personnel include a surgeon and an intensivist/anesthesiologist coordinating anesthesia care with veterinary staff. Necessary procedures include vascular access, monitoring ICP, managing hemodynamic disturbances during brain death, timekeeping, scribing, recording data, and obtaining samples.

There are several challenges with using the DBD model. While venous access is required for the induction and management of the animal outside the specifics of this model, additional vascular access can be challenging as it is our experience that the porcine vasculature is more prone to intimal dissection than human vessels. The use of ultrasound can be very helpful in obtaining access with less trauma to the vessels. The alternative to percutaneous approach is surgical cutdown with cannulation of the vessels under direct vision.

The identification of the cranial sutures is enhanced by using an electrosurgical pencil to cauterize the exposed skull's surface, resulting in the sutures being much more pronounced. Our experience with the intracranial balloon does not support the description in prior publications that the balloon could be advanced in a subdural position. It is technically challenging to enter the cranium without breaching the dura and it may be necessary to position the balloon in the subarachnoid or intraparenchymal space.^24^, ^44^

**FIGURE 3:**
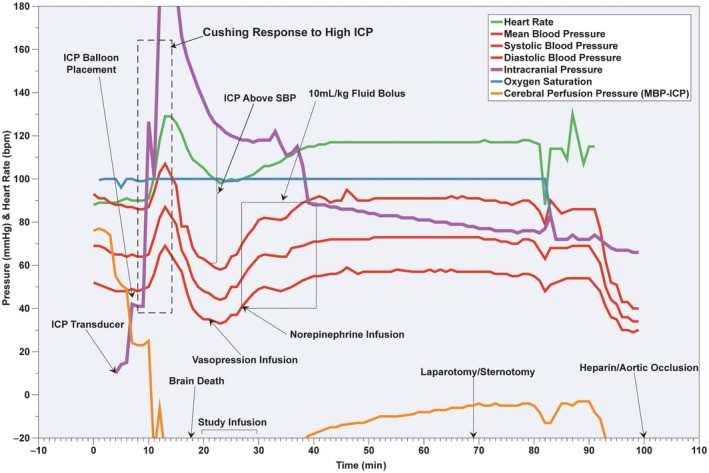
Event timeline and summary of hemodynamics during typical DBD procedure. Event timeline following vascular access during DBD procedure Pl(Section 2.2). ICP, intracranial pressure; MBP, mean blood pressure; SBP, systolic blood pressure. X‐axis: time (minutes) following vascular instrumentation.

Some authors suggest using the absence of oculo‐pupillary and corneal reflexes as confirmatory criteria for brain death.^53^ The interpretation of these tests in a paralyzed animal is very unreliable and the time required to fully allow the return of neuromuscular function can be lengthy. Loss of the Cushing reflex response in the presence of elevated ICP (above MAP) and the corresponding decrease of heart rate and blood pressure can be used to determine the start of brain ischemia (Figure 3). Keeping the intracranial pressure greater than mean arterial pressure for at least 30 min ensures the brain remains without blood supply and inevitably leads to irreversible brain structural damage. In our opinion, the confirmatory tests are not necessary since non‐perfused brain and brainstem cannot survive. This is supported by observation of Chen et al. who recorded a flat‐line electroencephalogram after 8–20 min after inflation of the balloon in every subject in their brain death model. The average time for all brainstem reflexes (including corneal, pupillary, oculomotor, and breathing reflexes) to disappear varied in each animal and generally occurred at an average time of 13.3 ± 1.5 min after balloon inflation. It is noteworthy that our initial experiments involving the establishment of brain death were performed in non‐paralyzed animals. These animals had significant seizure and tonic–clonic motion when the intracranial balloon was inflated. This created difficulties in maintaining stable vascular access.

The hemodynamic stability of the animals following brain death could be very challenging to manage. Initial attempts to maintain mean arterial pressure with volume and phenylephrine (ease of acquisition and low cost) may not always be successful. The additional infusion of vasopressors and inotropic agents is invariably necessary to maintain adequate hemodynamics (Figure 3 and Table 5).

Organ recovery within 1 hour from the diagnosis of brain death does not mirror clinical practice in which usually many hours pass in between declaration of brain death and organ procurement. We believe that this difference between clinical and research scenarios is not significant since the evidence suggests that hemodynamic, metabolic, and hormonal changes that are being attributed to the donor myocardial injury occur as early as 15 min after brain death.^54^

Another limitation is the potential impact of brain death kinetics (velocity) on post‐transplantation outcomes. Using an allotransplantation porcine model, Kerforne et al.^43^ demonstrated that renal primary graft function was less satisfactory in pigs grafted with kidneys procured after slow‐induced brain death as opposed to rapid‐induced brain death. This is in keeping with the observation that heart transplant recipients who receive organs from donors after traumatic brain injury (rapid‐induced) experience reduced mortality, rejections and cardiac allograft vasculopathy in comparisons to recipients who received organs from donors after non‐traumatic brain death (slow‐induced).^55^

### 4.2 Donation after circulatory death

This model is technically very demanding and requires advanced surgical and wire skills to minimize animal loss. When *in‐situ* recovery is planned, some technical nuances need to be considered. For example, occlusion of the head/neck vessels before initiation of *in‐situ* organ perfusion significantly impacts monitoring devices placed on cranial structures or inserted into neck vessels. The presence of lower extremity monitors and access is important.

Porcine tissues (as opposed to humans) are very friable, and the porcine ascending aorta is short. This poses some technical challenges when handling the tissues and inserting the catheters. The pressure‐volume (PV) loop catheter is extremely difficult to advance across the aortic valve into the LV. We found that positioning the sheath directly into LV allows a more straightforward path for the catheter across the valve. When *in‐situ* reperfusion of organs is planned, the head and neck vessels must be occluded before initiation of perfusion to preserve an uninterrupted process of brain ischemia after the flow to the thoracic and abdominal organs is restored. This requires dissection around the carotid artery and the occlusion of the vessel around the PV loop catheter.

Monitoring vital signs after the occlusion of the head and neck vessels presents difficulties, as many of the common clinical monitors and access points are using these vessels. Monitoring of the pulse oximetry cannot be accomplished from the head, ears, snout, etc. and must be placed caudally. Additionally, the venous access via the ear vein will no longer function and therefore cannot be used. It is important to establish venous and arterial access that permits use throughout the experiment, generally through the femoral vessels. Some animals may demonstrate refractory ventricular arrhythmia that is difficult to control with antiarrhythmic medications and external defibrillation may be required. Manipulation of the heart during placement of the sheaths and catheters must be done carefully as the cardiac function may be significantly impaired by this process.

A major limitation of the hypoxic circulatory arrest model of DCD is an unpredictable length of agonal period, i.e. the time interval from the withdrawal of respiratory support until circulatory arrest. From our experience, the agonal time in paralyzed animals ranges between 5 and 8 min. There is less variability in the agonal time when using animal models in comparison to clinical practice since paralyzed animals lack residual respiratory drive. To obtain consistent hypoxic intervals for all DCD study animals, it may be necessary to implement a variable period of no‐touch time following circulatory arrest prior to delivery of cardioplegia or initiation of *in‐situ* reperfusion.

**FIGURE 4:**
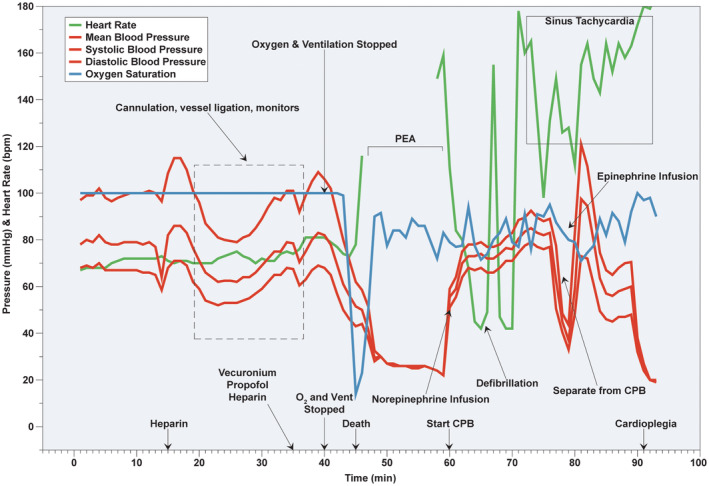
Event timeline and summary of hemodynamics during typical DCD procedure. Event timeline following vascular access during DCD procedure. CPB, cardiopulmonary bypass; PEA, pulseless electrical activity. X‐axis: time (minutes) following vascular instrumentation.

It is currently not known whether the porcine heart has the same tolerance for warm and cold ischemia as the human heart. Experience from the pig xenotransplant experiments showed that non‐ischemic porcine heart preservation (as opposed to cold static storage) is crucial for the survival of the transplanted hearts.^51^, ^56^, ^57^ This suggests that pig hearts might be more susceptible to cold ischemic injury than human hearts. Whether this difference in tolerance to cold ischemia between humans and pigs also applies to warm ischemia is currently not known (Figure 4).

## 5 ALTERNATIVE ANIMAL MODELS

### 5.1 Donation after brain death

An alternative to using a Foley catheter to increase intracranial pressure is the infusion of autologous blood in the brain parenchyma through a catheter placed in the subdural space.^27^ In this model, animals developed the typical Cushing reflex between 60 and 90 min after the start of the intracranial blood infusion (1.5 mL/min). Cooper et al. described a model in which brain death was induced by clamping or ligation of both carotid and subclavian arteries. When compared to the method of the sudden increase of intracranial pressure with the balloon, both models showed identical hemodynamic changes.^58^

### 5.2 Donation after circulatory death

Martin et al.^45^ described a large animal model of circulatory arrest by means of exsanguination. However, this mode of death does not reflect a real clinical DCD situation. Additionally, there is some evidence that death from asphyxiation causes more myocardial damage than death from exsanguination.^48^, ^59^

An alternative to the hypoxic pulmonary arrest DCD model is to achieve organ hypoperfusion through a combined pharmacological and technical approach to decrease mean arterial pressure.^30^, ^50^ Although such alternative models are very reliable and reproducible, they do not account for the effect of hypoxia, specifically hypoxic pulmonary vasoconstriction, on the physiologic changes in thoracic organs during the agonal period.^29^

## 6 CONCLUSIONS AND FUTURE PERSPECTIVES

Large animal models of DBD and DCD organ recovery are highly important to translational research groups working on strategies to optimize ischemia–reperfusion injury and organ preservation. They also provide an opportunity to facilitate and improve recovery technique, team technical skills, communication, and collaboration during innovative surgical procedures employed in organ recovery such as *in‐situ* reperfusion after circulatory death. Models can also allow for concurrent and collaborative research programs involving thoracic and abdominal organs.

Porcine models of DBD and DCD organ donation have been described previously.^25^, ^30^, ^43^ The purpose of this article was to present an overview of both models with the emphasis on key conceptual and practical considerations. We outlined step‐by‐step description of procedural details, instrumentation, and pharmacopeia of current DBD and DCD models. We described common pitfalls, limitations, and challenges inherent to these procedures, and offered alternatives. We believe that this work will provide a reliable resource for reproducible pre‐clinical donor organ intervention research and enable new investigators to address unmet needs in transplant medicine.

## AUTHOR CONTRIBUTIONS

FH, SM, NM, KD, MM, LF, WT, TW, MA, and MU contributed to the conception and design of the work; FH, SM, NM, KD, MM, LF, RS, and MU contributed to data acquisition; FH, SM, NM, and MU contributed to data analysis and interpretation; FH, SM, NM, and MU wrote the draft manuscript; FH, SM, NM, MM, LF, and MU revised the manuscript; all authors approved the final version for publication.

## Funding INFORMATION

This study was funded by a Team Seed Grant (#26685) from the University of Nebraska Collaborative Initiative Program; S. Merani, P.I.

## CONFLICT OF INTEREST STATEMENT

The authors declare that there is no conflict of interest regarding the publication of this article.

## ETHICS STATEMENT

The experiments and animal procedures were approved by the University of Nebraska Medical Center Institutional Animal Care and Use Committee (19‐133‐12‐FC, 21‐074‐10‐EP).
